# RACIAL RESIDENTIAL SEGREGATION AND SUICIDAL BEHAVIOR ACROSS THE LIFECOURSE AMONG BLACK AMERICANS

**DOI:** 10.1093/geroni/igae098.1366

**Published:** 2024-12-31

**Authors:** Eskira Kahsay, Briana Mezuk

**Affiliations:** University of Michigan, Ann Arbor, Michigan, United States; University of Michigan, Ann Arbor, Michigan, United States

## Abstract

Residential segregation is a well-studied contextual factor with adverse impacts on physical health. However, the neighborhood is both a material and social space. Given hypothesized mechanisms of increased social support and reduced discrimination, racial/ethnic homogeneity may not be necessarily harmful to mental health. Still, little is known about its implications for suicidality among Black Americans, particularly older adults. This study investigated the associations between residential segregation and suicidal behavior using the National Survey of American Life (N = 5,191; mean age: 42.5). Lifetime suicidal behavior (ideation [10.4%] and attempt [3.6%]) were self-reported. The Getis-Ord Gi* statistic was used to measure the spatial segregation of Black residents at the census-tract level (low, medium, high). Weighted mixed-effects models were fit, adjusting for demographics and tract-level poverty and stratified by age group (early [18-29], middle [30-49], older adulthood [50+]). The prevalence of ideation and attempt did not significantly differ across segregation levels. Odds of ideation were not significantly different in high vs low segregation areas though were significantly lower in medium vs low segregation areas (OR = 0.70, 95% CI: 0.49, 0.99). Findings for suicide attempt were non-significant. Among older adults, the odds of attempt were significantly lower for high vs low segregation areas (0.98, 95% CI: 0.97, 0.99) only. No significant findings were found for other age groups, except ideation in medium vs low segregation in early adults (OR = 0.33, 95% CI: 0.15, 0.74). Despite economic disadvantage in racially segregated areas, findings suggest higher segregation is not associated with increased suicidality.

